# A Fas^hi^ Lymphoproliferative Phenotype Reveals Non-Apoptotic Fas Signaling in HTLV-1-Associated Neuroinflammation

**DOI:** 10.3389/fimmu.2017.00097

**Published:** 2017-02-14

**Authors:** Soraya Maria Menezes, Fabio E. Leal, Tim Dierckx, Ricardo Khouri, Daniele Decanine, Gilvaneia Silva-Santos, Saul V. Schnitman, Ramon Kruschewsky, Giovanni López, Carolina Alvarez, Michael Talledo, Eduardo Gotuzzo, Douglas F. Nixon, Jurgen Vercauteren, David Brassat, Roland Liblau, Anne Mieke Vandamme, Bernardo Galvão-Castro, Johan Van Weyenbergh

**Affiliations:** ^1^Department of Microbiology and Immunology, Rega Institute for Medical Research, Clinical and Epidemiological Virology, KU Leuven, Leuven, Belgium; ^2^Department of Microbiology, Immunology & Tropical Medicine, The George Washington University, Washington, DC, USA; ^3^LIMI, Gonçalo Moniz Research Center (CPqGM), Oswaldo Cruz Foundation (FIOCRUZ), Salvador, Brazil; ^4^Bahiana School of Medicine and Public Health, Salvador, Brazil; ^5^Instituto de Medicina Tropical Alexander von Humboldt, Universidad Peruana Cayetano Heredia, Lima, Peru; ^6^Departamento de Enfermedades Infecciosas, Tropicales y Dermatológicas, Hospital Cayetano Heredia, Lima, Peru; ^7^INSERM UMR1043 and Pôle des Neurosciences, Hôpital Purpan, Université de Toulouse, Toulouse, France; ^8^Center for Global Health and Tropical Medicine, Unidade de Microbiologia, Instituto de Higiene e Medicina Tropical, Universidade Nova de Lisboa, Lisbon, Portugal

**Keywords:** Fas/CD95, proliferation, HTLV-1-associated myelopathy/tropical spastic paraparesis, lymphoproliferative disease, apoptosis, interferon, NF-κB, multiple sclerosis

## Abstract

Human T-cell lymphotropic virus (HTLV)-1 was the first human retrovirus to be associated to cancer, namely adult T-cell leukemia (ATL), but its pathogenesis remains enigmatic, since only a minority of infected individuals develops either ATL or the neuroinflammatory disorder HTLV-1-associated myelopathy/tropical spastic paraparesis (HAM/TSP). A functional *FAS* -670 polymorphism in an interferon (IFN)-regulated STAT1-binding site has been associated to both ATL and HAM/TSP susceptibility. Fas^hi^ T stem cell memory (Tscm) cells have been identified as the hierarchical apex of ATL, but have not been investigated in HAM/TSP. In addition, both *FAS* and *STAT1* have been identified in an IFN-inducible HAM/TSP gene signature, but its pathobiological significance remains unclear. We comprehensively explored Fas expression (protein/mRNA) and function in lymphocyte activation, apoptosis, proliferation, and transcriptome, in PBMC from a total of 47 HAM/TSP patients, 40 asymptomatic HTLV-1-infected individuals (AC), and 58 HTLV-1 -uninfected healthy controls. Fas surface expression followed a two-step increase from HC to AC and from AC to HAM/TSP. In HAM/TSP, Fas levels correlated positively to lymphocyte activation markers, but negatively to age of onset, linking Fas^hi^ cells to earlier, more aggressive disease. Surprisingly, increased lymphocyte Fas expression in HAM/TSP was linked to decreased apoptosis and increased lymphoproliferation upon *in vitro* culture, but not to proviral load. This Fas^hi^ phenotype is HAM/TSP-specific, since both *ex vivo* and *in vitro* Fas expression was increased as compared to multiple sclerosis (MS), another neuroinflammatory disorder. To elucidate the molecular mechanism underlying non-apoptotic Fas signaling in HAM/TSP, we combined transcriptome analysis with functional assays, i.e., blocking vs. triggering Fas receptor *in vitro* with antagonist and agonist-, anti-Fas mAb, respectively. Treatment with agonist anti-Fas mAb restored apoptosis, indicating biased, but not defective Fas signaling in HAM/TSP. *In silico* analysis revealed biased Fas signaling toward proliferation and inflammation, driven by RelA/NF-κB. Correlation of Fas transcript levels with proliferation (but not apoptosis) was confirmed in HAM/TSP *ex vivo* transcriptomes. In conclusion, we demonstrated a two-step increase in Fas expression, revealing a unique Fas^hi^ lymphocyte phenotype in HAM/TSP, distinguishable from MS. Non-apoptotic Fas signaling might fuel HAM/TSP pathogenesis, through increased lymphoproliferation, inflammation, and early age of onset.

## Key Points

- A two-step increase in cell death receptor Fas occurs upon HTLV-1 infection and disease progression.
- Unexpectedly, higher Fas level was linked to decreased cell death, increased lymphocyte proliferation/activation, and early disease onset.

## Introduction

Human T-cell lymphotropic virus 1 (HTLV-1) is an exogenous human retrovirus infecting 5–10 million people worldwide, mostly in HTLV-1 endemic regions (1). While a majority of HTLV-1 carriers remain asymptomatic (AC) lifelong, a minority (0.25–3%) progresses to either adult T-cell leukemia/lymphoma (ATL) or HTLV-1-associated myelopathy/tropical spastic paraparesis (HAM/TSP) (2, 3). Thirty years after its discovery, it is still enigmatic how a single retrovirus causes either fatal hematologic malignancy or neuroinflammation in a small subset of infected individuals. Among factors that allow to discriminate between the three clinical groups (AC, ATL, and HAM/TSP), humoral immunity (4) and the proteome (5, 6) have been described. In agreement with a role for immune activation (4, 6–9) in HAM/TSP pathogenesis, promising preclinical results were obtained with Jak kinase and NFκB inhibitors (10, 11). Very few drugs, e.g. valproate, have actually overcome the hurdle in transition from preclinical results (12) to clinical trial in HAM/TSP (13). Taken together, these studies point at a possible clinical benefit of decreasing lymphoproliferation and/or increasing apoptosis in HAM/TSP patients. HTLV-1-infected cells are driven toward spontaneous lymphoproliferation and oligoclonal expansion (14, 15). On the other hand, apoptosis (programmed cell death) is known to play a role in controlling lymphoproliferation in autoimmune diseases (16, 17). Fas (TNFRSF6/CD95/APO-1) is a death-domain-containing receptor of the tumor necrosis factor (TNF) receptor superfamily inducing apoptosis (17), when ligated by Fas ligand (FasL) or agonist antibodies (18). Fas-FasL signaling is proposed to play a role in both autoimmune and infectious diseases (17). In multiple sclerosis (MS) patients, increased Fas expression has since long been known (19), while resistance of T cells to Fas-mediated apoptosis has been linked to MS (20). In HTLV-1 infection, a wealth of data is available on pro- and anti-apoptotic effects of HTLV-1 infection, mainly its proto-oncogene tax (21). In the context of HAM/TSP immunopathogenesis, a role for Fas-FasL in the downregulation of immune response in the CNS has been suggested (22). Previous studies on Fas in HAM/TSP have shown increased levels of soluble Fas in serum (23, 24), and CSF (24), as well as surface expression in CD8 cells (25). A systems biology approach identified *FAS* (but not *FASL*) as a part of an interferon (IFN)-regulated gene signature in HAM/TSP patients (7). In addition, immunogenetic data revealed that a functional *FAS* -670 gene polymorphism is associated to both ATL (26) and HAM/TSP (27) disease susceptibility. Therefore, we hypothesized that lymphocyte Fas expression and/or apoptosis may reflect clinical status in HAM/TSP patients.

## Patients and Methods

**Figure 1:**
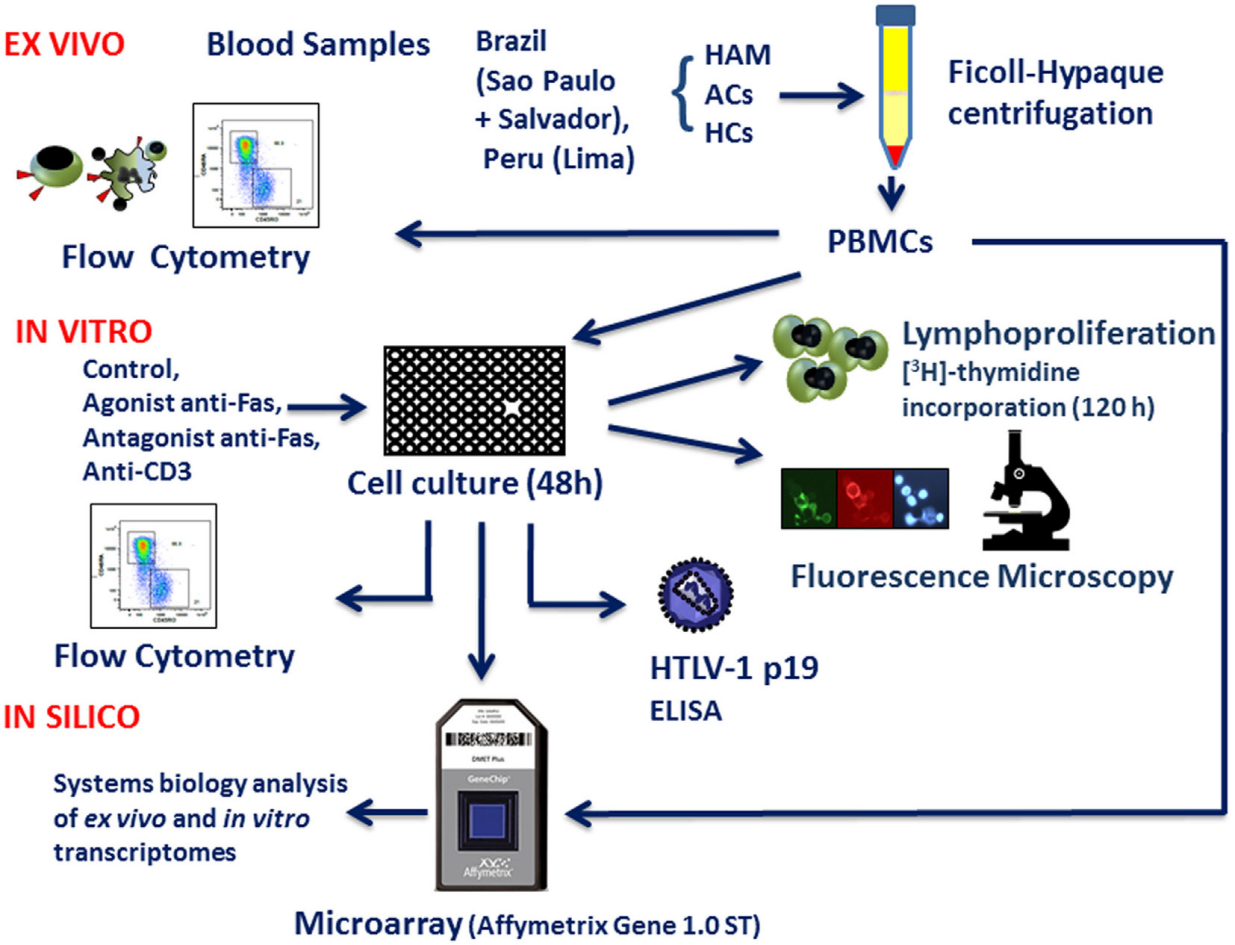
**Schematic representation of the methodology (*ex vivo, in vitro*, and *in silico* approaches)**.

A flow chart diagram (Figure 1) provides an overview of the study outline, cohorts, as well as *ex vivo, in vitro*, and *in silico* experimental approach, while patient information and sample use is summarized in Table 1.

**Table 1:** **Patient information and sample use**.

Patient	Age	Gender	Cohort	Analysis
1	NA	F	BA	*Ex vivo* flow cytometry

2	NA	M	BA	*Ex vivo* flow cytometry

3	NA	F	BA	*Ex vivo* flow cytometry

4	NA	F	BA	*Ex vivo* flow cytometry

6	NA	F	BA	*Ex vivo* flow cytometry

7	51	M	BA	*Ex vivo* and *in vitro* flow cytometry, *in vitro* apoptosis, *in vitro* lymphoproliferation

8	40	M	BA	*Ex vivo* flow cytometry, *in vitro* apoptosis, *in vitro* lymphoproliferation

9	40	F	BA	*Ex vivo* flow cytometry, *in vitro* apoptosis, *in vitro* lymphoproliferation

10	63	F	BA	*Ex vivo* flow cytometry, *in vitro* apoptosis, *in vitro* lymphoproliferation

11	51	F	BA	*Ex vivo* and *in vitro* flow cytometry, *in vitro* apoptosis, *in vitro* lymphoproliferation

12	36	M	BA	*Ex vivo* and *in vitro* flow cytometry, *in vitro* apoptosis, *in vitro* lymphoproliferation

13	40	F	BA	*Ex vivo* and *in vitro* flow cytometry, *in vitro* apoptosis, *in vitro* lymphoproliferation

14	60	F	BA	*Ex vivo* and *in vitro* flow cytometry, *in vitro* apoptosis, *in vitro* lymphoproliferation

15	44	M	BA	*Ex vivo* flow cytometry, *in vitro* apoptosis, *in vitro* lymphoproliferation

16	NA	F	BA	*Ex vivo* flow cytometry, *in vitro* apoptosis, *in vitro* lymphoproliferation

17	53	M	BA	*Ex vivo* and *in vitro* flow cytometry, *in vitro* apoptosis, *in vitro* lymphoproliferation, microarray

18	45	F	BA	*Ex vivo* and *in vitro* flow cytometry, *in vitro* apoptosis, *in vitro* lymphoproliferation, microarray

20	59	M	BA	*Ex vivo* and *in vitro* flow cytometry, *in vitro* apoptosis, *in vitro* lymphoproliferation

21	60	F	BA	*Ex vivo* and *in vitro* flow cytometry, *in vitro* apoptosis, *in vitro* lymphoproliferation

22	38	M	BA	*In vitro* lymphoproliferation

23	59	F	BA	*In vitro* lymphoproliferation

24	56	F	BA	*In vitro* flow cytometry, *in vitro* apoptosis, *in vitro* lymphoproliferation, microarray

25	49	F	BA	*In vitro* apoptosis

26	57	M	BA	*In vitro* apoptosis

27	49	F	BA	*In vitro* flow cytometry, *in vitro* apoptosis *in vitro* lymphoproliferation

28	60	M	BA	*In vitro* flow cytometry, *in vitro* apoptosis *in vitro* lymphoproliferation, microarray

29	46	M	BA	*In vitro* apoptosis, *in vitro* lymphoproliferation, microarray

31	50	M	BA	*In vitro* flow cytometry, *in vitro* apoptosis, *in vitro* lymphoproliferation, microarray

32	50	F	BA	*In vitro* flow cytometry, *in vitro* apoptosis, *in vitro* lymphoproliferation, microarray

33	62	F	BA	*In vitro* flow cytometry, *in vitro* apoptosis, *in vitro* lymphoproliferation

2,569	27	F	LI	*In vitro* apoptosis

2,570	50	F	LI	*In vitro* apoptosis

2,574	35	F	LI	*In vitro* apoptosis

2,817	64	F	LI	*Ex vivo* flow cytometry

2,819	32	F	LI	*Ex vivo* flow cytometry

2,821	63	F	LI	*Ex vivo* flow cytometry

2,822	50	F	LI	*Ex vivo* flow cytometry

2,823	64	M	LI	*Ex vivo* flow cytometry

SP5	32	F	SP	*Ex vivo* flow cytometry

SP6	65	F	SP	*Ex vivo* flow cytometry

SP7	62	F	SP	*Ex vivo* flow cytometry

SP8	47	F	SP	*Ex vivo* flow cytometry

SP26	35	M	SP	*Ex vivo* flow cytometry

SP30	72	M	SP	*Ex vivo* flow cytometry

SP32	27	M	SP	*Ex vivo* flow cytometry

SP36	52	F	SP	*Ex vivo* flow cytometry

SP46	61	F	SP	*Ex vivo* flow cytometry

*Cohorts: BA, Bahia; LI, Lima; SP, Sao Paulo*.

*NA, not available*.

HAM/TSP patients [*n* = 47, 66.0% female, mean age 50.2 ± 11.5 years, mean disease duration 5.6 ± 4.0 years (range 0.8–14 years), EDSS range 3–7 (mean 5.1 ± 1.2)] were recruited from three endemic regions (Sao Paulo and Salvador, Bahia, Brazil, and Lima, Peru) following written informed consent. Age- and gender-matched HTLV-1-infected asymptomatic carriers (AC, *n* = 40) and uninfected healthy controls (HC, *n* = 58) from the same endemic regions were included in the study. The study was approved by the Ethics Committees of University of Sao Paulo and FIOCRUZ-Bahia in Brazil and Universidad Peruana Cayetano Heredia in Lima, Peru. Diagnosis of HAM/TSP was according to WHO criteria (28) Antibodies to HTLV-1/2 were investigated by diagnostic ELISA (Murex, Abbott, Germany; Bioelisa HTLV-1 + 2, Biokit Spain) and confirmed by Western blot capable of discriminating between HTLV-1 and HTLV-2 (HTLV Blot 2.4, Genelab, Singapore). All HTLV-1-infected individuals were seronegative for HTLV-2 and HIV. For comparison with another neuroinflammatory disorder, data from MS patients [recruited during our previous study (29)] were used.

### Isolation of PBMC and *In Vitro* Cell Culture

PBMC isolated from 5–10 ml of heparinized venous blood by Ficoll–Hypaque density gradient centrifugation (Sigma-Aldrich) were washed twice with PBS and were plated in 24-well tissue culture plates (Costar, NY, USA) at 4 × 10^6^ cells/ml and incubated at 37°C and 5% CO_2_ in RPMI1640 medium supplemented with 2mM l-glutamine, gentamycin (50 µg/ml), and 10% heat-inactivated fetal calf serum (Gibco, NY, USA).

### HTLV-1 p19 and Proviral Load Quantification

Human T-cell lymphotropic virus-1 matrix protein p19 was quantified in cell-free supernatant of HAM/TSP patients’ PBMC and AC and HC using RetroTek HTLV-1/2 p19 Antigen ELISA kit (ZeptoMetrix) after 48 h of *in vitro* culture. Proviral load (PVL, i.e., viral DNA integrated into the host genome) in HAM/TSP patients and AC was quantified as published (30, 31).

### Quantification of Cell Surface Markers by Flow Cytometry

For phenotypic analysis, PBMC were resuspended at a density of 200,000 cells in 50 µl of 1% BSA, 0.1% NaN_3_ in PBS (+20% human serum to block Fc receptors), and incubated for 30 min on ice with mAbs specific for CD3, CD4, CD8, CD80, CD86, CD95/Fas, and HLA-DR and corresponding isotype controls (BD Biosciences). For total Fas surface quantification and apoptosis, a minimum of 100,000 events/sample were stained and acquired with FACSort and FACSCanto II flow cytometers (BD Biosciences) and analyzed using CellQuest and Diva software, respectively.

### Proliferation and Apoptotic Assays

Lymphoproliferation was quantified by [^3^H]-thymidine incorporation and flow cytometry [as described in Ref (29, 32)], the initial stage of apoptosis was analyzed using annexin V staining, whereas cells in the late/final stage of apoptosis were identified as a sub-diploid population by flow cytometry. Nuclear fragmentation was quantified by fluorescence microscopy and ELISA (Cell Death Detection plus, Boehringer Mannheim, Germany).

### Fas Triggering and Blocking Experiments

PBMC were cultured as above for 48 h in the presence or absence of agonist or antagonist anti-Fas mAbs (1 µg/ml, Alexis Biochemicals) or anti-CD3 mAb (Butantan Institute, Sao Paulo, Brazil) as a positive control for *in vitro* apoptosis.

### Microarray Analysis

Total RNA was extracted from PBMC according to manufacturer’s protocol (QIAgen, Venlo, The Netherlands). Whole genome microarray was performed at VIB Nucleomics (Leuven, Belgium) using GeneChip^®^ Human Gene1.0 ST Array (Affymetrix, Santa Clara, CA, USA), according to manufacturer’s specifications. Data were analyzed using Bioconductor limma package, using a moderated *t*-test, resulting *p*-values were corrected for genome-wide testing (5% FDR). All microarray raw data are available at Gene Expression Omnibus database (GEO, http://www.ncbi.nlm.nih.gov/geo/) series accession number GSE82160.

### Statistical Analysis

The use of parametric (*t*-test, Pearson correlation) or non-parametric (Mann–Whitney or Spearman rank correlation) tests was based on normal distribution as determined by Kolmogorov–Smirnov test (all GraphPad Prism v5.0 or v6.0). A *p*-value of <0.05 was considered significant for all statistical tests. Transcriptome-wide correlation of FAS mRNA expression levels was calculated using Spearman rank correlation test, with stringent correction for multiple testing (5% FDR).

## Results

### A Two-Step Increase in *Ex Vivo* Total Lymphocyte Fas Surface Expression in HTLV-1-Infected Individuals and HAM/TSP Patients, Distinguishable from MS Patients

**Figure 2:**
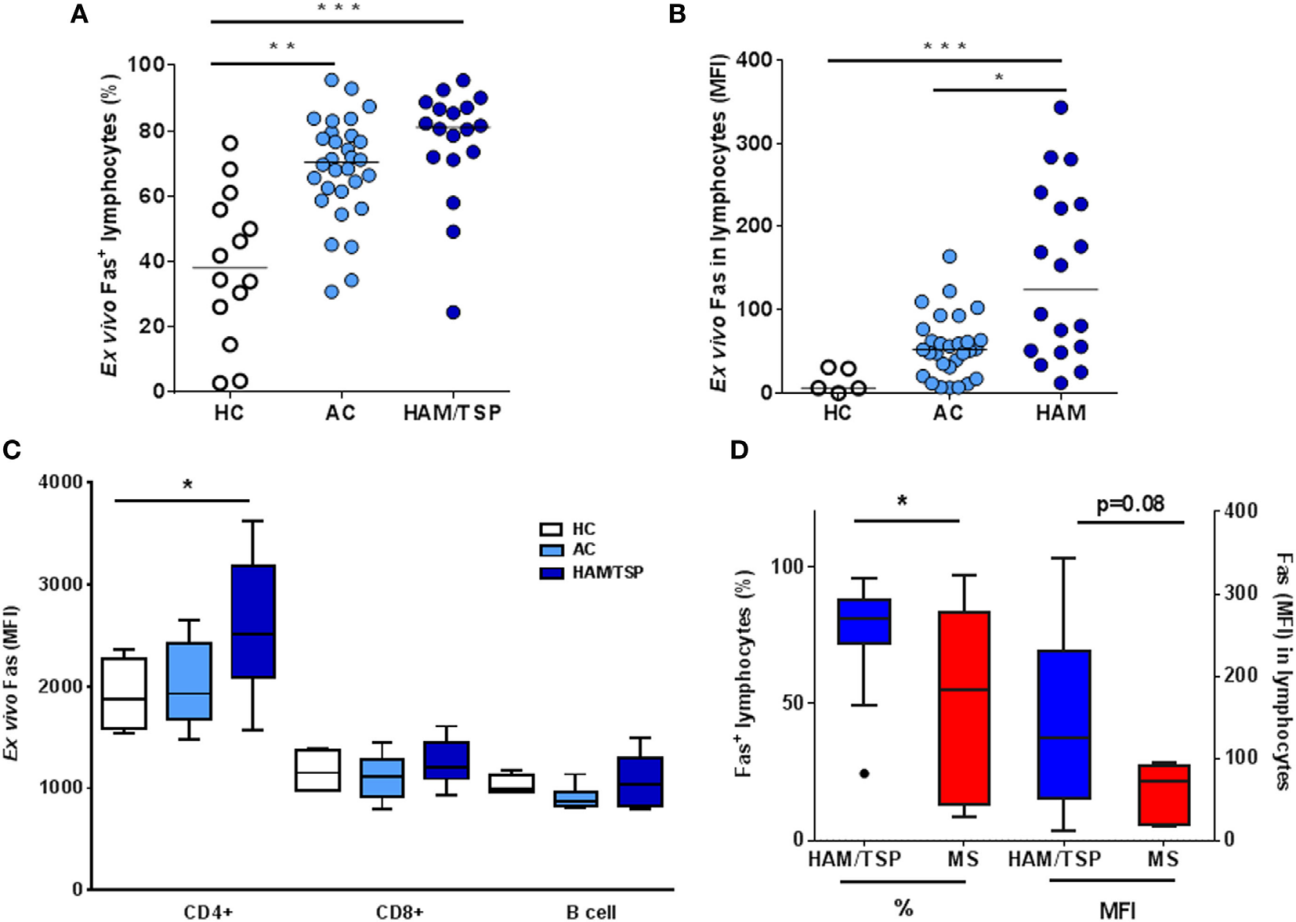
***Ex vivo* lymphocyte Fas surface expression in HTLV-1-infected individuals (AC), HAM/TSP, and multiple sclerosis (MS) patients**. Using flow cytometry, Fas levels as % **(A)** and mean fluorescence intensity (MFI, on a per cell basis) **(B)** were quantified in HC, AC, and HAM/TSP patients (*p < 0.05, **p < 0.01, ***p < 0.001; Kruskal–Wallis, with Dunn’s multiple comparison post-test). **(C)** Fas expression in CD4, CD8, and B cells was quantified in *ex vivo* PBMC in HC, AC, and HAM/TSP patients (ANOVA, *p* = 0.067, post-test for linear trend *p* < 0.05). **(D)** *Ex vivo* Fas levels (% and MFI) are compared between neuroinflammatory diseases HAM/TSP and MS (Mann–Whitney test, *p < 0.05).

In a first cohort, we quantified surface Fas levels as well as apoptosis by flow cytometry, *ex vivo* in PBMC from HC (HTLV-1-negative, *n* = 14), AC (HTLV-1-positive, *n* = 30), and HAM/TSP patients (*n* = 18). We observed a significant increase in *ex vivo* levels (%) of Fas^+^ lymphocyte in AC (1.8-fold) as well as in HAM/TSP patients (2.1-fold), when compared to HC (Kruskal–Wallis, Dunn’s posttest, *p* < 0.05, *p* < 0.001, respectively, Figure 2A). Moreover, lymphocyte Fas level on a per-cell basis, expressed as mean fluorescence intensity (MFI), revealed an eight-fold increase in AC and a striking 19-fold increase in HAM/TSP (Kruskal–Wallis, Dunn’s post-test, *p* < 0.001), when compared to HC, but also when compared to AC (*p* < 0.05, Figure 2B), indicating that clinical progression to HAM/TSP is characterized by a predominant Fas^hi^ lymphocyte population, possibly primed for apoptosis. To confirm the two-step model of Fas increase, we performed a *post hoc* test for linear trend, which was highly significant (*p* < 0.001) for both % (slope 18.8) and MFI (slope 64.1).

Next, we proceeded to examine Fas expression in CD4, CD8, and B cell subsets in more detail in an independent second cohort of HC (*n* = 7), AC (*n* = 6), and HAM/TSP patients (*n* = 9). There was no difference in the percentage of cells expressing Fas between the three clinical groups for either cellular subset (Figure 2C). However, we observed a small but significant linear trend in Fas MFI of CD4^+^ T cells with clinical status (ANOVA *p* = 0.067, post-test for linear trend *p* < 0.05, slope 349.2), but not in CD8^+^ T cells or B cells. Thus, the strongest difference between the clinical groups was in total Fas^+^ lymphocytes rather than specific subsets, revealing a Fas^hi^ phenotype in HAM/TSP. To verify if this Fas^hi^ phenotype might be shared among neuroinflammatory disorders, we compared Fas expression between HAM/TSP and MS patients. As shown in Figure 2D, we found a significant 1.6-fold increase in % of *ex vivo* Fas^+^ lymphocytes in HAM/TSP (Mann–Whitney, *p* = 0.03), as well as a 2.4-fold increase in Fas MFI, which approached statistical significance (Mann–Whitney, *p* = 0.08).

Finally, *ex vivo* spontaneous apoptosis in HAM/TSP and AC, as measured by DNA degradation (quantified as sub-diploid cells in flow cytometry) occurred at very low levels (<0.2% of PBMC, data not shown). Therefore, we questioned if the observed *ex vivo* increase in lymphocyte Fas surface expression in HAM/TSP reflected the immunological, virological, or clinical status of HAM/TSP patients, rather than an apoptosis-prone status.

### *Ex Vivo* Lymphocyte Fas Surface Expression Correlates to Immune Activation Markers in HAM/TSP

**Figure 3:**
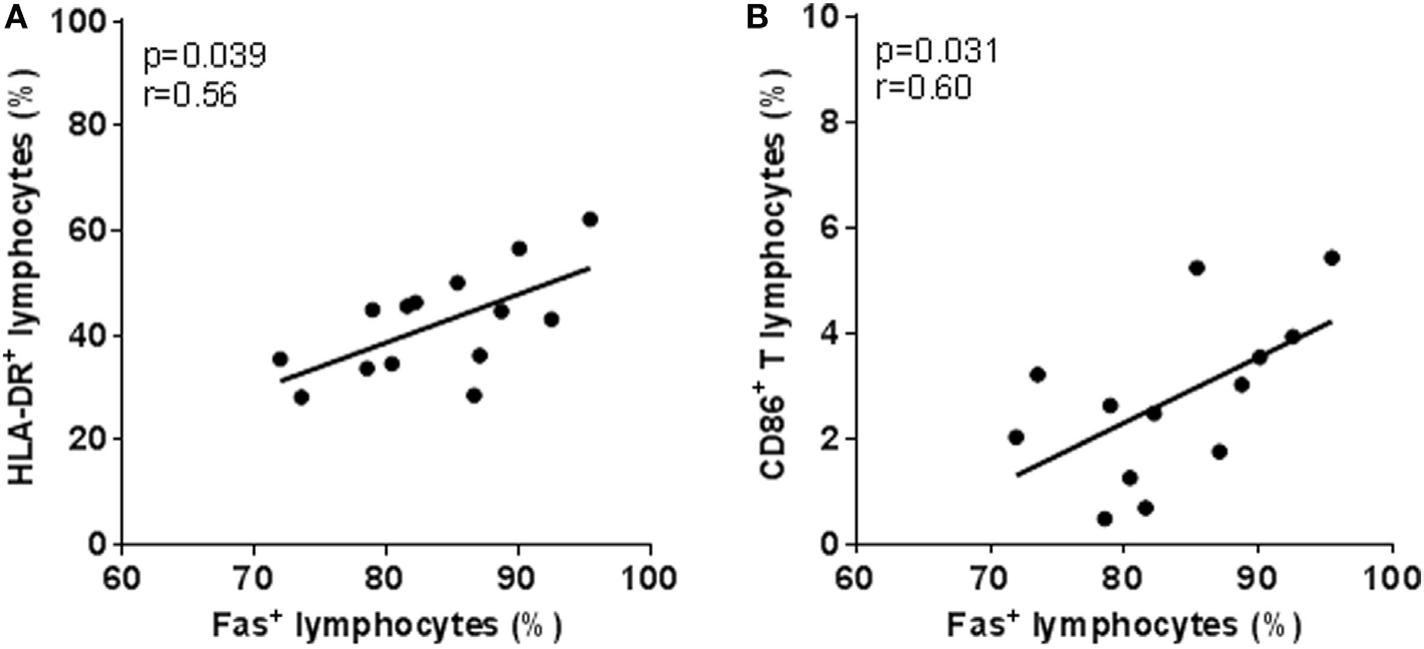
**Increased *ex vivo* lymphocyte Fas surface expression in HAM/TSP patients correlates with activation markers**. Positive correlation between the percentage of Fas^+^ lymphocytes and **(A)** HLA-DR^+^ (*p = 0.039, Spearman’s *r* = 0.56, *n* = 14) and **(B)** CD86^+^ (*p = 0.031, Spearman’s *r* = 0.60, *n* = 13) lymphocytes in HAM/TSP patients.

To explore possible clinical relevance of this increased lymphocyte Fas in HAM/TSP patients, we correlated *ex vivo* Fas surface expression to patient demographic and clinical data. We observed that, in HAM/TSP, *ex vivo* lymphocyte Fas (% or MFI) was not correlated to age, gender, disease duration, or severity. In addition, *ex vivo* lymphocyte Fas was not significantly correlated to PVL in AC or HAM/TSP (*p* > 0.05). However, *ex vivo* Fas levels (%) correlated significantly to lymphocyte activation markers HLA-DR and CD86 (Figures 3A,B), implying that increased Fas expression may be coupled to immune activation and/or inflammation in HAM/TSP.

### *In Vitro* Fas^+^ Lymphocyte Levels Correlate Negatively to Both Age of Onset and *In Vitro* Apoptosis: A Selective Defect in HAM/TSP Patients?

**Figure 4:**
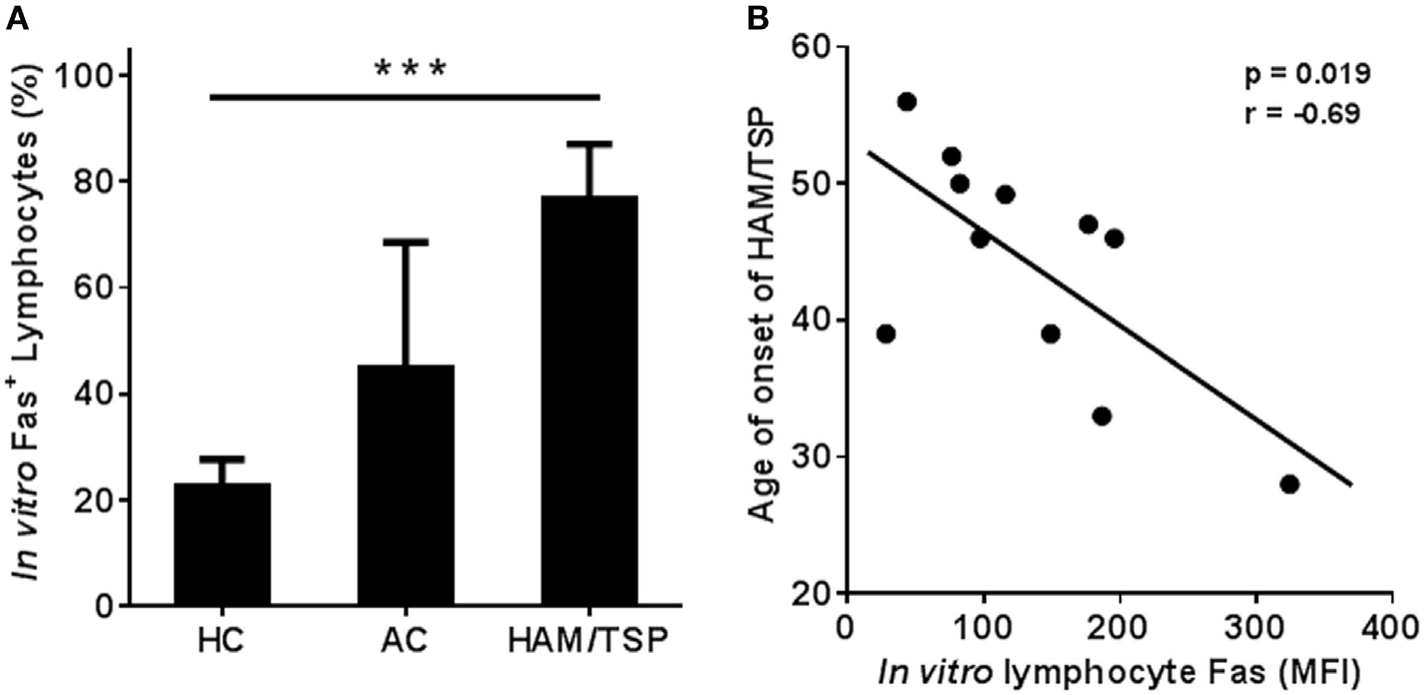
**Significant linear trend in Fas^+^ lymphocyte levels in PBMC of HC, AC, and HAM/TSP patients upon *in vitro* culture, and negative correlation with age of onset of HAM/TSP**. **(A)** Fas levels were quantified by flow cytometry after 48 h of *in vitro* culture. Fas^+^ lymphocytes (%) gradually increase (HC: *n* = 12; AC: *n* = 4; HAM: *n* = 12) upon infection (AC) and further upon disease progression to HAM/TSP (ANOVA, *p* = 0.0005; posttest for linear trend, *p* < 0.0001). **(B)** Lymphocyte Fas levels (after 48 h of *in vitro* culture) quantified by flow cytometry (MFI) correlate negatively to age of onset in HAM/TSP patients (*p = 0.019, Pearson’s *r* = −0.69, *n* = 11).

Upon quantification of *in vitro* Fas^+^ lymphocyte expression in HC, AC, and HAM/TSP patients by flow cytometry, we again observed a two-step increase in % Fas^+^ lymphocytes: two-fold in AC and 3.4-fold in HAM/TSP vs. HC (post-test for linear trend, *p* = 0.0001, slope 27.0) (Figure 4A). In HAM/TSP, *in vitro* Fas levels per-cell (MFI) were even more pronounced, with an eight-fold increase over HC. Hence, clinical status impacts both *ex vivo* (Figures 2A,B) and *in vitro* (Figure 4A) Fas expression. In addition, Fas *in vitro* levels showed a significant negative correlation to age of disease onset in HAM/TSP patients (*p* = 0.019, Pearson’s *r* = −0.69, *n* = 11) (Figure 4B), but not to age, disease duration, and gender, suggesting Fas^hi^ phenotype predisposes to earlier, aggressive disease manifestation. Further, *in vitro* Fas expression neither correlated to viral p19 protein level (*p* = 0.41), nor to PVL (*p* = 0.14) in HTLV-1-infected individuals (data not shown).

**Figure 5:**
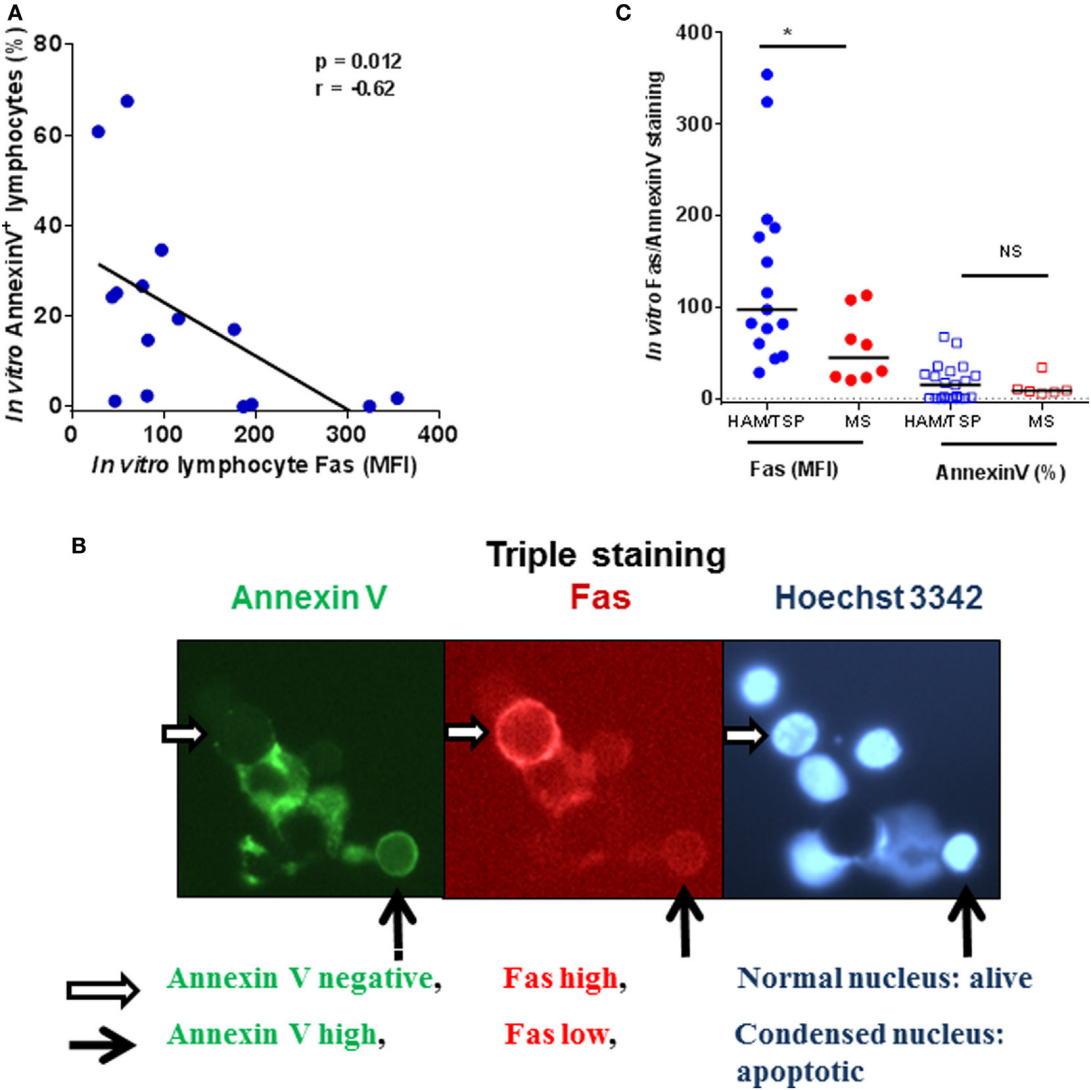
**Fas^hi^ cells are apoptosis-resistant in HAM/TSP patients**. **(A)** Fas mean fluorescence intensity (MFI, on a per-cell basis) negatively correlates to apoptosis (quantified as % annexin V^+^ cells) in lymphocytes of HAM/TSP patients (*p = 0.012, Spearman’s *r* = −0.63, *n* = 15). **(B)** In the middle panel is a representative image of a non-apoptotic Fas^hi^ cell (indicated by a white horizontal arrow). This Fas^hi^ cell is annexin V negative as visualized in the first panel and displays a normal nuclear morphology seen in the third panel. On the contrary, a Fas^lo^ cell in panel 2 (black vertical arrow), displays pronounced annexin V staining (panel 1) and is undergoing apoptosis, as evidenced by nuclear condensation, and is being engulfed by a macrophage (panel 3). **(C)** *In vitro* Fas levels (MFI) and apoptosis (% of Annexin V^+^ cells) are compared between neuroinflammatory diseases HAM/TSP and multiple sclerosis (Mann–Whitney test, *p < 0.05).

In agreement with its role as a death receptor in immune homeostasis, Fas surface expression positively correlates with spontaneous *in vitro* apoptosis in HC, while this correlation was lost in AC (data not shown). Surprisingly, *ex vivo* Fas expression correlated negatively (Figure S1 in Supplementary Material) to spontaneous *in vitro* apoptosis in HAM/TSP. Furthermore, *in vitro* Fas level (MFI) also correlates negatively to lymphocyte apoptosis in HAM/TSP (Figure 5A). This negative correlation was confirmed by fluorescence microscopy. As shown in Figure 5B, Fas^hi^ cells are negative for annexin V staining and display normal nuclear morphology, whereas Fas^lo^ cells were seen to undergo apoptosis by both annexin V staining and nuclear condensation/fragmentation, occasionally triggering phagocytosis by macrophages, emphasizing their apoptotic nature. Since resistance to Fas induced apoptosis has been observed *in vitro* in lymphocytes from MS patients (33), we compared *in vitro* lymphocyte Fas expression and apoptosis between HAM/TSP and MS patients. As shown in Figure 5C, there was a significant increase (2.4-fold, Mann–Whitney test, *p* = 0.019) in Fas MFI in HAM/TSP as compared to MS patients, but not apoptosis (as measured by annexin V staining, Mann–Whitney test, *p* = 0.84). In contrast to HAM/TSP, no correlation was observed between Fas MFI and apoptotic cells in MS patients (*p* = 0.35, data not shown). Taken together, the significant negative correlations between *ex vivo* and *in vitro* Fas lymphocyte expression and *in vitro* apoptosis observed only in HAM/TSP, suggest a possible selective defect in Fas-mediated apoptosis. Hence, we next aimed to comprehensively explore non-apoptotic Fas signaling in HAM/TSP.

### Fas Expression Positively Correlates to Lymphoproliferation *In Vitro* and *Ex Vivo* in HAM/TSP

**Figure 6:**
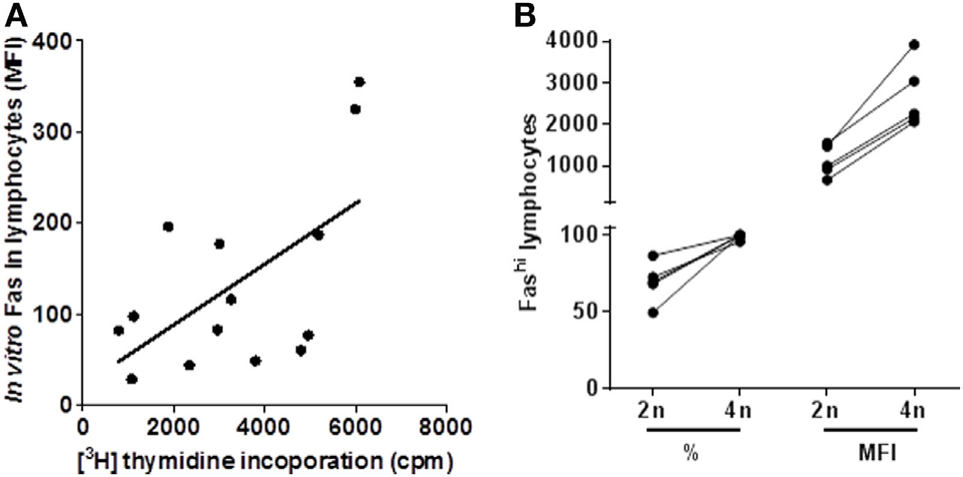
**Fas surface expression correlates positively with *in vitro* and *ex vivo* lymphoproliferation in HAM/TSP patients**. **(A)** *In vitro* Fas expression as measured by flow cytometry (MFI) correlates positively to lymphoproliferation quantified by [3H]-thymidine incorporation (*p = 0.018, Pearson’s *r* = 0.62, *n* = 14). **(B)** *Ex vivo* Fas surface expression measured by flow cytometry (% and MFI) is significantly higher in proliferating (tetraploid, 4*n*) cells vs. diploid (2*n*) cells in HAM/TSP patients (paired *t*-test, **p = 0.0082 and **p = 0.0023, respectively, *n* = 5).

We quantified *in vitro* spontaneous lymphoproliferation by [^3^H]-thymidine incorporation in HAM/TSP patients. Surprisingly, we found that Fas expression positively correlates to spontaneous lymphoproliferation *in vitro* (Figure 6A), which might imply that the observed defect in Fas-mediated proapoptotic signaling in HAM/TSP might be explained as a bias in Fas signaling toward proliferation rather than apoptosis. Therefore, we hypothesized that Fas^hi^ cells might be already proliferating *in vivo* in HAM/TSP although at a very low level. We thus extended our previously described (29) sensitive flow cytometry assay to quantify Fas^+^ diploid vs. tetraploid (proliferating) lymphocytes *ex vivo* in HAM/TSP patients, stained immediately after PBMC isolation, without *in vitro* culture. As shown in Figure 6B, virtually all of the proliferating cells were Fas^hi^ (99.2 ± 0.8%), as compared to non-proliferating lymphocytes (69.4 ± 5.9%, Paired *t*-test, *p* = 0.0082).

### Stimulation with Agonist Fas mAb *In Vitro* Can Trigger Apoptotic Signaling in HAM/TSP

**Figure 7:**
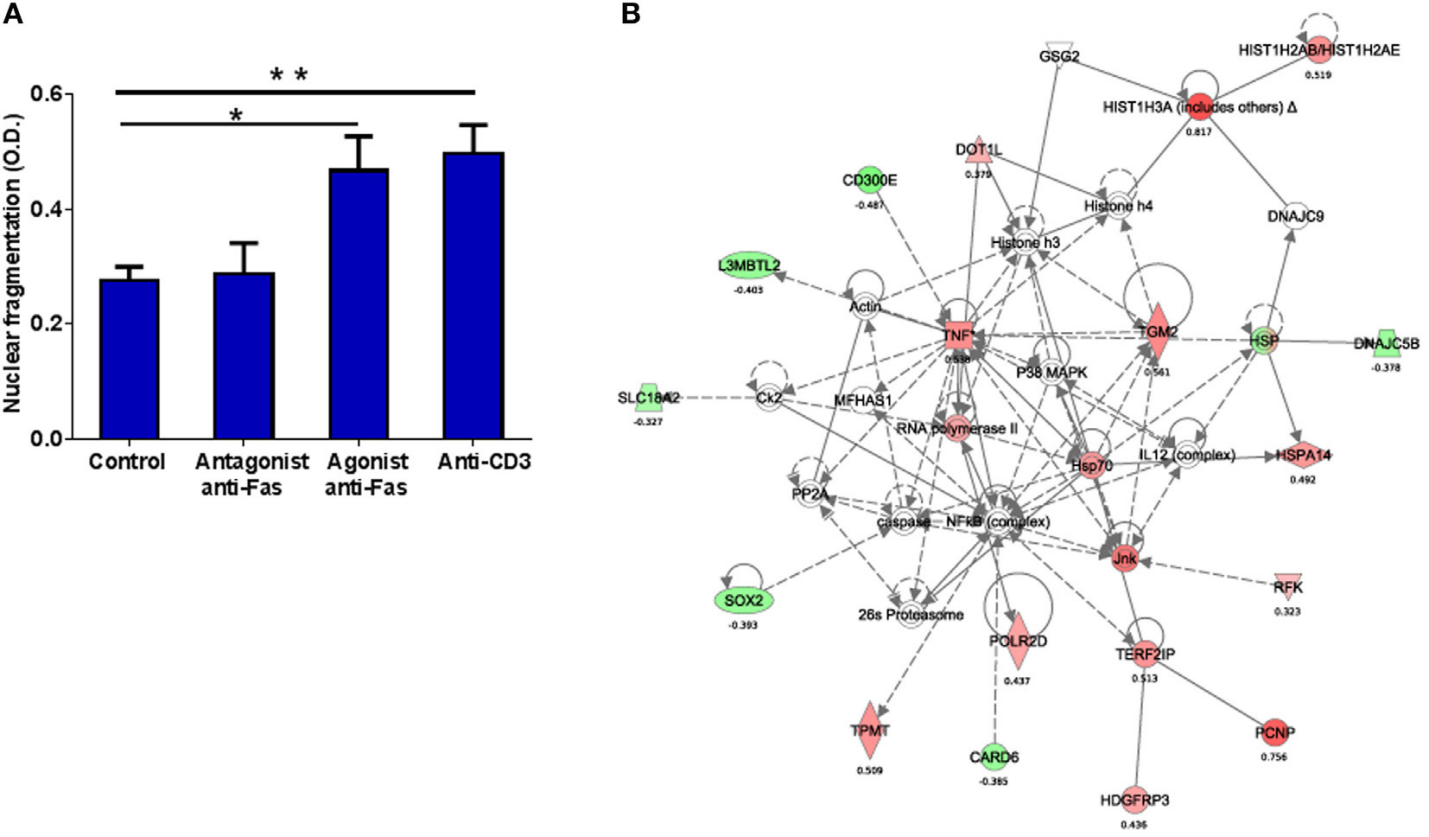
***In vitro* Fas triggering with agonist anti-Fas mAb induces apoptosis in HAM/TSP and activates a molecular network linking apoptosis, proliferation and inflammation**. **(A)** Agonist (ago) anti-Fas mAb, but not antagonist (ant) anti-Fas mAb increased apoptosis (quantified by CellDeathPlus ELISA) in PBMC upon *in vitro* treatment for 24 h when compared to control (untreated) PBMC. Treatment with anti-CD3 mAb was used as a positive control (ANOVA, with Bonferroni’s post-test *p < 0.05, **p < 0.01). **(B)** Top molecular network (score = 34, linking cell-to-cell signaling, interaction, and cellular growth and proliferation) identified by Ingenuity pathway analysis (IPA) among 249 genes significantly up- and downregulated (red and green, respectively) in PBMC of HAM/TSP patients by *in vitro* treatment with agonist anti-Fas mAb.

We then examined if this apparent defect in Fas-mediated apoptosis might be reversible by stimulating with agonist anti-Fas mAb, and if blocking with antagonist anti-Fas mAb could reveal ongoing Fas-FasL signaling in HAM/TSP. Hence, we treated PBMC *in vitro* with anti-Fas mAb (agonist or antagonist) or anti-CD3 mAb as a positive control. No decrease in spontaneous apoptosis was observed upon treatment with antagonist anti-Fas mAb, confirming our hypothesis of inactive Fas-FasL signaling *in vitro* in HAM/TSP. Interestingly, treatment with agonist anti-Fas mAb resulted in significantly increased apoptosis (1.7-fold, *p* < 0.05), similar to treatment with anti-CD3 mAb (positive control, 1.8-fold, *p* < 0.01) (Figure 7A). These results imply that agonist anti-Fas mAb treatment can restore the apparent defect in apoptosis in HAM/TSP, at least *in vitro*.

### Systems Analysis of Gene Expression Profiles Upon Fas Triggering vs. Fas Blocking in HAM/TSP

Considering the significant correlation between *in vitro* Fas expression to age of onset in HAM/TSP, we resorted to genome-wide transcriptional analysis of PBMC treated *in vitro* with agonist or antagonist Fas mAb, to explore the broad pro/antiapoptotic, inflammatory, proliferative, and immunoregulatory Fas signaling pathways specifically triggered in HAM/TSP. Microarray analysis revealed that *in vitro* treatment with agonist anti-Fas mAb, significantly downregulated 190 genes and upregulated 59 genes (Tables S1A,B in Supplementary Material), while treatment with antagonist anti-Fas mAb downregulated 38 genes and upregulated 18 genes (Tables S1C,D in Supplementary Material). Thus, triggering Fas signaling effects a broader gene spectrum than inhibiting it. This was also evident from Ingenuity^®^ pathway analysis (IPA), since no biological functions were significantly associated with antagonist anti-Fas mAb treatment, whereas treatment with agonist anti-Fas mAb resulted in 22 significantly associated biological functions (5% FDR-adjusted and a stringent cut-off of at least five enriched molecules per pathway) (Table S2 in Supplementary Material). The top 10 biological functions activated by agonist anti-Fas mAb (Table S2 in Supplementary Material), highlight cellular migration, especially of myeloid cells. In addition, IPA network analysis (Figure 7B) of Fas-triggered gene expression reveals a central role for NFκB pro-survival signaling, connecting several upregulated proliferative and inflammatory molecules (TNF, JNK, RNA Polymerase II, POLR2D, HIST1H3A, HIST1H2AB) as well as downregulated anti-proliferative genes (L3MBTL2, CARD6). This central role for NFκB signaling was confirmed by ingenuity upstream regulator analysis, identifying RelA as the top upstream regulatory molecule upon triggering Fas signaling (target genes: BCL2A1, CASR, CXCL3, ICAM1, L3MBTL2, PTGES, TGM2, TNF, and TPMT; *p* = 0.000032). Again, blocking Fas signaling did not yield any significantly enriched upstream regulators (using the same stringent cut-off of five enriched molecules/pathway, data not shown).

### Genome-Wide Correlation of *Ex Vivo* Fas RNA Levels in HAM/TSP Confirms a Significant Association to Proliferation but Not Apoptosis

Finally, we used a pathway-based data mining approach, to test our hypothesis of biased Fas signaling, and to possibly extend our findings by including additional pro- and anti-apoptotic genes (e.g., TRAIL, cFlip, etc.). For this purpose, we explored possible interactions of Fas mRNA within the *ex vivo* global gene expression profile in PBMC of HAM/TSP patients (*n* = 6). Using transcriptome-wide correlation, 4,554 genes significantly correlated to Fas transcript levels (Table S3 in Supplementary Material), after stringent FDR-correction for multiple testing. Using annotated ingenuity pathways, we found a significant enrichment for proliferation-related genes (159 of 4,554 genes, *p* = 0.023). However, apoptosis, as defined by IPA, was not enriched amongst the *ex vivo* Fas-correlating genes (71 genes out of 4,554 genes, *p* = 0.10).

## Discussion

In this study, we combined *ex vivo, in vitro*, and systems analysis of Fas expression with functional apoptosis and proliferation assays, thereby providing an all-inclusive approach of the biological and clinical relevance of Fas signaling in HAM/TSP. We observed a two-step increase in *ex vivo* Fas expression: first, a greater percentage of Fas^+^ lymphocytes upon HTLV-1 infection and second, a strong increase in expression of the death receptor at the single-cell level upon HAM/TSP disease progression. In addition, for the first time, we demonstrate that Fas expression correlates negatively to apoptosis and age of onset, but positively to immune activation and lymphoproliferation.

The most surprising finding of this study is a selective defect in Fas-mediated apoptosis in HAM/TSP patients. First, both *ex vivo* and *in vitro* Fas levels negatively correlated to *in vitro* apoptosis (Figure 5A; Figure S1 in Supplementary Material). Second, by fluorescence microscopy (Figure 5B), we document that Fas^lo^, but not Fas^hi^ cells, preferentially undergo apoptosis *in vitro*. Third, *in vitro* treatment of PBMC with agonist anti-Fas mAb, but not antagonist anti-Fas mAb, was able to trigger apoptosis and restore the selective defect in HAM/TSP patients. Fourth, *in silico* analysis of the HAM/TSP transcriptome revealed a large number of transcripts (>4,500) significantly correlating to Fas mRNA level, but are not enriched for apoptotic pathways. Taken together, our data indicate that the death receptor is fully functional in HAM/TSP, and not in a dormant state, but skewed towards other biological pathways. Similar to our observation in HAM/TSP, increased Fas (19) and resistance to Fas-triggered apoptosis (34) has been reported in MS, which was also supported by gene expression profiling (35). Nevertheless, our data reveal that the Fas^hi^ phenotype is HAM/TSP-specific, since Fas expression was increased both *ex vivo* and *in vitro*, as compared to MS patients. Strikingly, the increase in non-apoptotic Fas receptor is also negatively correlated to age of disease onset in HAM/TSP (Figure 4B), rendering Fas as a clinically relevant molecule. It should be stated, however, that formal demonstration of the possible clinical utility of Fas expression or Fas downstream signaling targets as biomarker(s) in HAM/TSP will require confirmation of our findings in prospective cohort studies with a long-term clinical follow-up. In addition, agonist anti-Fas mAb, although restoring the defect in apoptosis in HAM/TSP, would not be a therapeutic option given that anti-Fas mAb therapy caused liver injury and lethality in mice (36). In the absence of clinical benefit of antiretrovirals in HAM/TSP, immunomodulatory options include IFN-α/β, glucocorticoids, cyclosporine, and ascorbic acid (32, 37, 38). We previously demonstrated that IFN-β can restore defective B cell CD86 upregulation in HAM/TSP (29). As in MS, defective Fas-mediated apoptosis in HAM/TSP patients may be overcome by IFN-β therapy (39, 40). In addition to IFN therapy, our *in silico* analysis might reveal novel treatment options. As shown in Figure 7B, a molecular network that elegantly describes the interplay between the molecular players of apoptosis (CARD6, caspases), proliferation (POLR2D, L3MBTL2), and inflammation (TNF, JNK), with a central role for NFκB. Therefore, our data confirm and extend the findings of Oh et al. (11) and Talledo et al. (9) who pointed at the importance of NFκB signaling in HAM/TSP from a pharmacological and immunogenetic perspective. Furthermore, our Fas-triggered gene expression in HAM/TSP reveals the same upstream regulator (Rel A), which is associated to active disease in MS (35). Thus, transcriptomics can reveal neuroinflammatory disorders sharing analogous biological pathways, indicating approved MS drugs to be considered in HAM/TSP, but also allow the identification of possible novel therapeutic targets, e.g., TGM2 or L3MBTL2 (Figure 7B).

**Figure 8:**
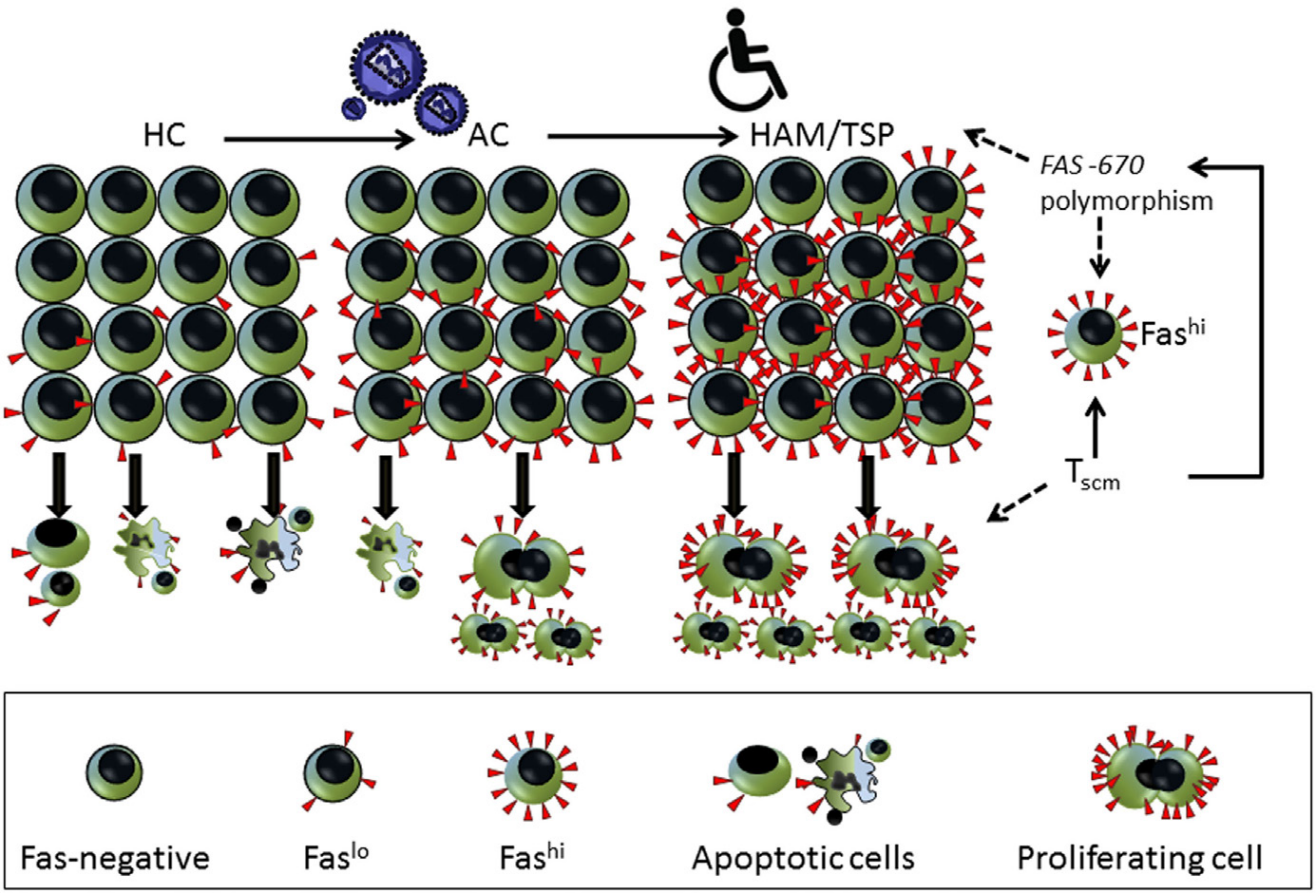
**Model indicating the two-step increase in *ex vivo* lymphocyte Fas surface expression**. First, following human T-cell lymphotropic virus (HTLV)-1 infection, there is an increase in lymphocyte Fas expression (%) in AC. Second, upon progression to HAM/TSP, Fas expression is increased on a per-cell basis as mean fluorescence intensity (MFI) (Figures 2A,B). In agreement with its role as a death receptor, Fas^+^ cells in HC are primed to follow the apoptotic pathway, depicting nuclear condensation and cell blebbing, which is lost upon HTLV-1 infection (AC). In contrast, in HAM/TSP patients, Fas^hi^ cells are driven toward proliferation (Figures 6A,B). We recently discovered a genotype/phenotype interaction for the *FAS* -670 polymorphism with both apoptosis and proliferation in adult T-cell leukemia (ATL) patients and healthy controls (Khouri et al., submitted). This Fas^hi^ proliferating and chemotherapy-resistant leukemic phenotype is in agreement with the recently discovered CD4 T stem cell memory (Tscm) hierarchical apex of ATL. The same *FAS* -670 polymorphism also determined CD4 Tscm levels in a genome-wide twin study, confirming our hypothesis (Khouri et al., submitted). Therefore, a genetically determined interferon (IFN)/STAT1/FAS axis might help explain the proliferative, non-apoptotic phenotype in HAM/TSP suggesting CD4 Tscm as a pivotal factor not only in ATL but also in HAM/TSP pathogenesis. Considering STAT1 and FAS are in the HAM/TSP gene signature, our data further refine the data of Tattermusch et al. (7) It is not unexpected that a Tscm phenotype is absent from the disease signature, since Tscm are rare (2–3%) (45) and their genome-wide expression profile is intermediate between naïve and central memory T cells. However, Tscm cells have a Fas^hi^, apoptosis-resistant, and drug-resistant, proliferative phenotype, in agreement with their stem cell-like nature. Interestingly, the proliferating cells in HAM/TSP patients were almost exclusively Fas^hi^ (Figure 6B), compatible with a Tscm phenotype.

Regarding HAM/TSP pathogenesis, both genetic and environmental triggers have been suggested (41). Interestingly, in a large cohort in the same endemic area (Salvador, Bahia), a city with Afro-descendent demography, probable (but not definite) HAM/TSP occurred in 31% of AC during 8-year follow-up (42), which suggests lifetime risk in this population is 10-fold higher than previously reported (41). As for environmental factors, co-infection with gram-positive bacteria, as in infective dermatitis, has been shown to trigger early HAM/TSP in children from the same endemic area (43, 44). Concerning genetics, a single *FAS* -670 polymorphism has been associated to both ATL (26) and HAM/TSP (27) susceptibility. Since this polymorphism also determined CD4 T stem cell memory (Tscm) levels in a genome-wide twin study (Khouri et al., submitted), the proliferative, non-apoptotic Fas^hi^ cells in HAM/TSP are reminiscent of a Tscm phenotype (45), as outlined in Figure 8. However, since CD4 or CD8 Tscm represent only a minor subset of Fas^+^ lymphocytes (46), a Tscm origin of Fas^hi^ cells is not likely, considering the two-step increase we observed both *ex vivo* and *in vitro* (Figures 2A,B and 4A), first in AC and second in HAM/TSP.

Non-apoptotic Fas signaling toward proliferation has been previously demonstrated (47, 48), while tax gene expression and cell cycling, but not cell death, are selected during HTLV-1 infection *in vivo* (49). Tax mediates its antiapoptotic activity by activating the NFκB pathway (50), associating NFκB to cell survival and inflammation, similar to our *in silico* findings. In addition, Tax-deregulated autophagy and cFLIP expression are responsible for resistance to apoptosis *in vitro* (51), in agreement with our *ex vivo* and *in vitro* results. In contrast, many viral infections are associated with heightened apoptosis. The most striking example is HIV (52), which manipulates apoptotic pathways to enable efficient viral replication (53). In the case of HTLV-1, *in vitro* culture triggers viral protein synthesis and subsequent cytokine-driven lymphoproliferation (14). However, Fas did not correlate to PVL, similar to (25) and two other published cohorts (*p* > 0.5 for test and training sets) (7). Interestingly, PVL also did not correlate to apoptosis or age of disease onset, in contrast to Fas. A previous larger study with sufficient statistical power also demonstrated PVL does not correlate to age of onset in HAM/TSP (54). Furthermore, viral p19 protein levels did not correlate to Fas in our cohort. Taken together, increased Fas levels in HAM/TSP appear to be driven by an IFN/STAT1 axis, either genetically (27) or environmentally (43) linked, rather than by the virus itself, suggesting that the role of Fas in HAM/TSP pathogenesis is independent of PVL. Therefore, it is tempting to speculate that a similar IFN/STAT1 signaling pathway might underlie the suggested deleterious role of CD80^+^ B cells, correlating positively to disease severity, also independent of PVL (29).

In conclusion, our results suggest that defective Fas-mediated apoptosis is linked to early disease onset and might be an additional factor in HAM/TSP pathogenesis, independent of PVL. Triggering Fas signaling, rather than inhibiting it, induces a specific gene set with a central role for NFκB pro-survival signaling. Thus, our integrated *ex vivo, in vitro, in silico* approach identifies biased pro-inflammatory and proliferative Fas signaling in HAM/TSP, revealing possible novel therapeutic targets.

## Author Contributions

JVW designed research; SMM, FEL, TD, Ricardo K, DD, GS-S, GL, and JVW performed research; SVS, DFN, JV, and AMV contributed to data analysis; FEL, Ramon K, CA, MT, EG, DB, RL, and BGC provided patient samples; SMM and JVW analyzed data and wrote the paper.

## Conflict of Interest Statement

The authors declare that the research was conducted in the absence of any commercial or financial relationships that could be construed as a potential conflict of interest. The reviewer MM and handling Editor declared their shared affiliation, and the handling Editor states that the process nevertheless met the standards of a fair and objective review.
