# Supplementary material for: A Fashi Lymphoproliferative Phenotype Reveals Non-Apoptotic Fas Signaling in HTLV-1-Associated Neuroinflammation
Source: Front Immunol. 2017 Feb 14;8:97. doi: 10.3389/fimmu.2017.00097 (PMC5306374; doi:10.3389/fimmu.2017.00097)
Supplement: Supplementary file 1 [file data_sheet_1.docx]

Supplementary Figure

**1**

**Supplementary Figure. 1. Fas *ex vivo* surface expression negatively predicts subsequent *in vitro* apoptosis in HAM/TSP patients.** In HAM/TSP PBMCs, *ex vivo* lymphocyte Fas level (mean fluorescence intensity, MFI) correlates negatively to subsequent *in vitro* lymphocyte apoptosis measured by AnnexinV staining (*p=0.028, Spearman r= -0.59, n=14).

Supplementary Tables (Excel document)

Supplementary Table 1: List of significantly up-regulated and down-regulated genes upon treatment with agonistic anti-Fas mAb and antagonistic anti-Fas mAb.

Supplementary Table 2: List of all significant biological functions upon treatment with agonist Fas mAb. Twenty-two biological functions were significant with a stringent cut-off of at least 5 molecules in the pathway. (FDR<0.05).

Supplementary Table 3: List of 4554 transcripts that significantly correlate to Fas transcript after correction for genome-wide testing (FDR <0.05)
